# Trends in Bullying Victimization and Social Unsafety for Sexually and Gender Diverse Students

**DOI:** 10.1007/s10964-024-01943-6

**Published:** 2024-01-25

**Authors:** W. J. Kiekens, R. Van der Ploeg, J. N. Fish, T. Salway, T. M. L. Kaufman, L. Baams

**Affiliations:** 1 Department of Sociology/Interuniversity Center for Social Science Theory and Methodology, (ICS), University of Groningen, Grote Rozenstraat 31, 9712 TG Groningen, The Netherlands; 2 Department of Pedagogy and Educational Sciences, University of Groningen, Grote Rozenstraat 38, 9712 TJ Groningen, The Netherlands; 3 Department of Family Science, School of Public Health, University of Maryland, 4200 Valley Dr., College Park, MD 20740 USA; 4 Faculty of Health Sciences, Simon Fraser University, 8888 University Drive, Burnaby, BC V5A 1S6 Canada; 5 Department of Education and Pedagogy, Utrecht University, Heidelberglaan 1, 3584 CS Utrecht, The Netherlands

**Keywords:** Sexual orientation, Gender diversity, Bullying victimization, Social safety, Adolescents

## Abstract

Research has documented trends in bullying victimization for sexually diverse adolescents in the US, but trends regarding school social unsafety are understudied and there is a dearth of research examining these trends for gender diverse adolescents. This study aimed to identify disparities in bullying victimization and feelings of social unsafety in schools for sexually and gender diverse adolescents. Data stem from the 2014 (*N* = 15,800; *M* age = 14.17, *SD* = 1.50), 2016 (*N* = 22,310; *M* age = 14.17, *SD* = 1.49), and 2018 (*N* = 10,493; *M* age = 14.02, *SD* = 1.52) survey cycles of the Social Safety Monitor, a Dutch cross-sectional school-based study. Findings indicate that sexual orientation disparities remained relatively small, but stable over time, while gender diverse adolescents remained more likely to be victimized and feel unsafe in school, with larger disparities overall. Monitoring these trends is highly relevant, especially considering recent negative developments regarding societal acceptance of sexual and gender diversity.

## Introduction

Research has shown sexual orientation- (Martin-Storey & Fish, 2019) and gender identity-based (Martín-Castillo et al., 2020) disparities in bullying victimization. These experiences can lead to poorer mental health outcomes, such as depressive symptoms (Mongelli et al., 2019) or suicidality (de Lange et al., 2022). Recently, research has proposed that the absence of social safety, understood as the availably of protective ties resulting in reliable social connection, belongingness, inclusion, recognition, and protection, may also affect sexually and gender diverse adolescents’ mental health (Diamond & Alley, 2022). Social safety may depend on the social context (Diamond & Alley, 2022) and considering the salience of schools in adolescents’ lives, it is imperative to study experiences with social unsafety in schools, where research has identified sexual orientation- (Mooij, 2016) and gender identity-based (Snapp et al., 2015) disparities as well. Considering positive changes in societal acceptance of sexual and gender diversity in the past decades (Huijnk, 2022), it is necessary to examine whether the presence and size of these disparities has changed over time. However, the current research on trends in bullying victimization disparities is US-focused, does not consider trends of social safety in schools, and has not examined trends for gender diverse adolescents. Therefore, the present study aimed to identify trends in bullying victimization and social unsafety in schools for sexually and gender diverse adolescents.

### Social Acceptance and the Developmental Collision Hypothesis

In the past decades, acceptance of sexual and gender diversity has increased in Western countries. For example, in the Netherlands, positive attitudes toward sexually diverse people have increased, although this trend plateaued from 2017 onwards (Huijnk, 2022). There has been a similar improvement in social attitudes of acceptance of gender diversity, albeit with lower prevalence of these positive attitudes overall. Research has also shown improved societal acceptance throughout Europe (Huijnk, 2022; ILGA Europe, 2023). A different indicator of the acceptance of sexual diversity is the rising number of countries that have legalized same-sex marriage or implemented other protective laws and policies (Human Rights Campaign, 2022). These changes in societal acceptance should theoretically affect the experiences of sexually and gender diverse people in a positive way. For instance, it could be expected that positive societal attitudes are related to decreased sexual orientation- and gender modality-based disparities in bullying victimization and reduced feelings of social unsafety in schools.

However, research in the US suggests that disparities have remained stable or even widened amid growing societal acceptance. This paradox reflects a potential clash between normative and sexual orientation and gender modality-specific developmental processes in the context of social change (Russell & Fish, 2016, 2019). That is, amid growing acceptance of sexual and gender diversity, adolescents “come out” at younger ages than previous generations (Russell & Fish, 2016); the mean age at which sexually diverse people disclosed their sexual orientation has declined (Bishop et al., 2020), from 21 years old in 1979 to 14 years old in 2015 in the US (Russell & Fish, 2016). This younger age of disclosure now coincides with a developmental period during which peer social regulation and the policing of gender expression and heteronormative behavior is heightened (Russell & Fish, 2019). The developmental collision hypothesis (Russell & Fish, 2019) posits that, despite the improved societal acceptance, sexually and gender diverse adolescents may remain vulnerable – or maybe are now more vulnerable – to bullying victimization and feel unsafe in school (Russell et al., 2014).

### Trends in Bullying Victimization and School Social Unsafety for Sexually and Gender Diverse Adolescents

Research has examined changes in disparities in bullying victimization for sexually diverse adolescents, but not gender diverse adolescents. For example, data from high school students in Massachusetts from 1999 to 2013 showed that average rates of bullying victimization decreased over time (especially for boys), but sexual orientation-based disparities remained stable in size (Goodenow et al., 2016). Similarly, a national US study examined changes in victimization from 2009 to 2017 and showed an overall decrease in general victimization, but sexually diverse adolescents remained at an increased risk of victimization relative to their heterosexual peers (Poteat et al., 2019).

Less research has examined whether there are changes in disparities in school social unsafety for sexually diverse adolescents, and none have examined this for gender diverse adolescents. An exception is a US-based study that found that rates of skipping school because of feeling unsafe decreased over the years (especially for boys), but sexual orientation-based disparities remained stable in size (Goodenow et al., 2016). Further, a Dutch study found an increase in sexual orientation-based disparities in school social unsafety between 2008 and 2010 (Mooij, 2016). Gaining insight into (trends in) disparities of students’ feelings of school social unsafety is important because a lack of social safety produces a constant vigilance for threats in the environment which can lead to perseverating, fearfulness, and social isolation (Diamond & Alley, 2022) and ultimately poorer mental health (Kiekens et al., 2022). Feelings of social unsafety might depend on the social context (Diamond & Alley, 2022) and considering the saliency of schools in adolescents’ lives, it is imperative to study experiences with social unsafety in schools, which could be a distinct target for intervention programs or school policies.

## Current Study

Research on trends in bullying victimization disparities is US focused, does not consider trends of social unsafety in schools, and has only focused on sexual-orientation-based trends and not on gender modality-based trends. Therefore, the present study aimed to identify trends in bullying victimization and social unsafety in schools for sexually and gender diverse adolescents in the Dutch context. Following the developmental collision hypothesis (Russell & Fish, 2019) and previous research on trends in victimization for sexually diverse adolescents (Goodenow et al., 2016; Poteat et al., 2019), several expectations about disparities within each year and about trends over time were formulated for this study. Regarding disparities within each year, it was expected that sexually diverse adolescents (measured as romantic attraction) would report more bullying victimization and feelings of school social unsafety than heterosexual adolescents. Similarly, it was expected that gender diverse adolescents (measured as gender modality) would report more bullying victimization and feelings of school social unsafety than cisgender adolescents. Regarding trends over time, it was expected that sexual orientation- and gender modality-based disparities in bullying victimization and school social unsafety remained stable over time.

## Methods

### Procedures and Participants

Data stem from the 2014 (Sijbers et al., 2014), 2016 (Scholte et al., 2016), and 2018 (Nelen et al., 2018) survey cycles of the Social Safety Monitor. These are national cross-sectional school-based studies on the social safety of students in the Netherlands. Data collection was commissioned by the Dutch Ministry of Education, Culture, and Science as schools are required by law to monitor social safety in schools. All middle and high schools in the Netherlands were approached to participate, but school participation was voluntary. Participating schools were asked that students be seated behind separate desks when administering the survey. Participation for students was voluntary, data collection was anonymous, and no individual login codes were used. The Ethics Committee of the Sociology Department of the University of Groningen approved the use of the data and advised, to protect participants’ privacy, to conduct analyses at the aggregated (school) level. Yet, analyses in the present study were conducted at the individual (student) level because the research questions concern individual level outcomes. However, results are only reported at the mean level and data will not be shared, both to protect participants’ privacy and in line with the advice of the Ethics Committee.

Fifty-six middle and high schools participated in 2014, 91 middle and high schools participated in 2016, and 34 middle and high schools participated in 2018. This amounts to *N* = 15,800 students in 2014 (*M* age = 14.17, *SD* = 1.50), *N* = 21,310 students in 2016 (*M* age = 14.17, *SD* = 1.49), and *N* = 10,493 students in 2018 (*M* age = 14.02, *SD* = 1.52). Of note, in 2018, *n* = 2550 students in special secondary education completed a shortened survey where not all constructs of interest were measured; these students were therefore omitted. Because of the biannual nature of the Social Safety Monitor, *n* = 23 schools participated in two or three survey cycles. Because no individual login codes were used, it was impossible to track students across survey cycles. All schools and students were included in the present study irrespective of whether schools participated across multiple cycles. Descriptive statistics by survey cycle are presented in Table 1.

**Table 1:** Descriptive Statistics by Survey Year

Variables	2014 *(N* = 15,800)	2016 (*N* = 21,310)	2018 (*N* = 10,493)	
	*M* (*SD*)/%	*M* (*SD*)/%	*M* (*SD*)/%	*n* missing
Verbal victimization	53.73%	49.46%	47.53%	1041
Material victimization	30.54%	26.85%	28.16%	1162
Social victimization	35.82%	31.60%	32.78%	1317
Light physical victimization	24.29%	21.92%	25.05%	1404
Heavy physical victimization	14.89%	13.50%	10.37%	1472
Sexual victimization	12.85%	11.80%	13.92%	765
School social unsafety^1^	1.97 (0.81)	1.90 (0.77)	1.81 (0.71)	
Sexual orientation				6044
Other-gender attracted	79.37%	78.44%	78.49%	
Same or both-gender attracted	20.63%	21.56%	21.51%	
Gender modality				4939
Cisgender	92.55%	93.54%	97.12%	
Gender diverse	7.45%	6.46%	2.88%	
Level of education				2913
Practical training and special education	10.74%	14.04%	5.44%	
Prevocational education/pre-[applied] university education	89.26%	85.96%	94.56%	
Grade^2^	2.42 (1.21)	2.50 (1.23)	2.64 (1.33)	
Age	14.17 (1.50)	14.16 (1.49)	14.02 (1.52)	6
Migration background				6152
No migration background	67.71%	77.86%	87.55%	
Migration background	32.29%	22.14%	12.45%	
Sex assigned at birth				3547
Male	50.15%	54.31%	50.05%	
Female	49.85%	45.69%	49.95%	

*Notes.* ^1^Answer options were 0 = Very safe, 1 = Safe, 2 = Neither safe nor unsafe, 3 = Unsafe, and 4 = Very unsafe

^2^Grade ranged from grade 1 to grade 6

### Measures

#### Bullying Victimization

Across all survey cycles, participants were presented 28 questions about victimization experiences in school from the end of the summer holiday until the questionnaire was filled out (February-April of the same school year). Different types of victimization were assessed that were based on established types of bullying victimization (Olweus, 1996). That is, verbal victimization (4 questions; e.g., “You were called names”), material victimization (5 questions; e.g., “Your belongings were scratched or damaged”), social victimization (6 questions; e.g., “You were ignored, others acted as if you were not there”), light physical victimization (5 questions; e.g., “You were purposefully bumped in to or hurt”), heavy physical victimization (4 questions; e.g., “Others started a fight you”), and sexual victimization (4 questions; e.g., “Others made sexual remarks towards you”) were assessed. Answer options were 0 = *Never*, 1 = *Less than once per month*, 2 = *Once per month or more*, 3 = *Once per week or more*, and 4 = *Once per day or more*. For every type of victimization, mean scores were calculated (Cronbach’s α verbal victimization = 0.87; α material victimization = 0.90; α social victimization = 0.88; α light physical victimization = 0.93; α heavy physical victimization = 0.89; α sexual victimization = 0.83). Because the distribution of the victimization variables was skewed (e.g., half of participants did not experience bullying victimization), original responses were dichotomized to participants who were never or occasionally victimized (0) and participants who were victimized monthly, weekly, or daily (1) (Solberg & Olweus, 2003).

#### School Social Unsafety

Across all survey years, participants were asked, “Could you indicate how safe you feel at school?” with answer options 0 = *Very unsafe*, 1 = *Unsafe*, 2 = *Neither safe nor unsafe*, 3 = *Safe*, and 4 = *Very safe*. This was recoded so that higher scores indicate feelings of unsafety in school.

#### Sexual Orientation

Romantic attraction was used as a measure for sexual orientation. Across all years, romantic attraction was assessed by asking, “I could fall in love with a girl” and “I could fall in love with a boy” with answer options ranging from 1 = *Completely agree* to 5 = *Completely disagree*. In line with previous research using the Social Safety Monitor (Kaufman & Baams, 2022), a dummy variable was created based on this question and a question on the participant’s sex assigned at birth (“What is your sex?,” 0 =*Male*, 1 = *Female*). That is, when they checked response options *completely agree*/*agree* with the question on falling in love with the other-gender and *completely disagree/disagree* with falling in love with the same gender, they were categorized as 0 = *Other-gender attracted*. When participants checked response options *completely disagree/disagree* with falling in love with the other-gender and *completely agree*/*agree* with the question on falling in love with the same gender; or when they *completely agree*/*agree/neither agree nor disagree* with falling in love with the same gender and *completely agree*/*agree/neither agree nor disagree* with falling in love with the other gender, they were categorized as 1 = *Same or both-gender attracted*.

#### Gender Modality

Across all years, participants were asked, “Do you identify as a boy?” and “Do you identify as a girl?” with answer options 0 = *Yes, completely*, 1 = *Partly*, and 2 = *No*. Together with a question on participants’ sex assigned at birth, a dummy variable was created, similar to a previous study using the Social Safety Monitor (Kaufman & Baams, 2022). When participants’ gender modality and sex assigned at birth aligned (e.g., sex is male, *completely* agrees with identifying as a boy, *no* agreement with identifying as a girl), they were categorized as 0 = *Cisgender*. When participants’ gender modality and sex assigned at birth did not align (e.g., sex is male, *completely* agrees with identifying as a boy, *completely*/*partly* agreement with identifying as a girl; *partly/no* agreement with identifying as a boy, *completely*/*partly*/*no* agreement with identifying as a girl), they were categorized as 1 = *Gender diverse*.

#### Survey Year

Two dummy variables were created, one that reflected 0 = *Participated in 2014* and 1 = *Participated in 2016* and one that reflected 0 = *Participated in 2014* and 1 = *Participated in 2018*.

#### Covariates

Several covariates were used. Because the Dutch secondary education system differentiates between several educational levels (Nuffic, 2023), level of education was controlled for (“What type of education do you follow” recoded as 0 = *Practical training and special education*, 1 = *Prevocational education/pre-[applied] university education*). For migration background, participants were asked in which country their mother and father were born. If both parents were born in the Netherlands, this was coded as 0 = *No migration background*. If at least one parent was born outside of the Netherlands, it was coded as 1 = *Migration background*. Sex assigned at birth and grade (ranging from grade 1 to grade 6) were also controlled for.

### Analytical Approach

Trend analyses were conducted separately for sexual orientation and gender modality. All analyses were conducted using STATA 17 (StataCorp, 2021). The data had a nested structure (students nested in schools) which was accounted for in the analyses by using the *svy* command. Missing data on all variables was imputed using the chained equation option (StataCorp, 2023) taking the nested structure into account. Results from analyses using list wise deletion and using multiple imputation yielded similar results. The analyses concerning bullying victimization were conducted in a logistic regression framework because all measures were dichotomous. As school social unsafety was an ordinal measure with 5 categories, these analyses were conducted in a linear regression framework (Norman, 2010). Analyses for sexual orientation were stratified by sex assigned at birth; those for gender modality were not.

Trend analyses were conducted in several steps (Homma et al., 2016). First, as descriptive analyses, the prevalence of bullying victimization and school social unsafety by sexual orientation and gender modality group by year were examined. This gave an initial (descriptive) picture of the size of the disparities in bullying victimization and school social unsafety over the survey years.

Second, for each survey year, sexual orientation and gender modality-based differences in bullying victimization and school social unsafety were estimated. These analyses were adjusted for level of education, grade, migration background, sexual attraction (only in models with gender modality as predictor), and gender modality (only in models with sexual orientation as predictor). As the analyses for gender modality were not sex-stratified, sex assigned at birth was also adjusted for. These analyses allowed to test for sexual orientation and gender modality-based disparities in bullying victimization and feelings of school social unsafety.

Last, the *survey year by sexual orientation* and *survey year by gender modality* interaction terms were examined. These analyses allowed to assess whether disparities in bullying victimization and school social unsafety have changed over time and, if so, if they either widened or narrowed. Such interaction is needed because odds ratios from different samples cannot be directly compared. The interaction terms compare the odds of bullying victimization or school social unsafety for a specific group (e.g., same or both-gender attracted) in a specific year (e.g., 2016) relative to the reference groups (e.g., other-gender attracted) to the odds of those with the same identity (e.g., same or both-gender attracted) relative to the reference group (e.g., other-gender attracted) in the comparison year (e.g., 2014). Put differently, the interaction terms produced “odds of odds ratios” (OOR). When ORR had a value above 1.00, it indicated a widening disparity, whereas a value below 1.00 indicated a narrowing disparity. Regarding the analyses in a linear regression framework with school social unsafety as outcome variable, an estimate above 0 indicated a widening disparity and an estimate below 0 indicated a narrowing disparity.

## Results

Table 2 displays descriptive disparities in bullying victimization and school social unsafety by sexual orientation across survey years and Table 3 does this by gender modality. Among males, regardless of sexual orientation, verbal, material, social, light physical, and sexual victimization first declined and then increased over the years. Heavy physical victimization and school social unsafety declined over all years. Among females, a mixed picture was observed. That is, verbal, material (only for same-gender attracted females), social, light physical (only for same-gender attracted females), and heavy physical victimization declined over the years, as did school social unsafety. Material (only for other-gender attracted females), light physical (only for other-gender attracted females) and sexual victimization (only for other-gender attracted females) declined and then increased over the years. Sexual victimization remained stable over the years but only for same-gender attracted females. Overall, sexual orientation-based differences were relatively small.

**Table 2:** Bullying Victimization and School Social Unsafety across Survey Years by Sexual Orientation

	2014		2016		2018	
	Proportion	95% CI	Proportion	95% CI	Proportion	95% CI
**Males**						
*Verbal victimization*						
Other-gender attracted	0.55	[0.54, 0.57]	0.52	[0.50, 0.54]	0.54	[0.51, 0.57]
Same or both-gender attracted	0.60	[0.56, 0.64]	0.54	[0.51, 0.56]	0.57	[0.52, 0.61]
*Material victimization*						
Other-gender attracted	0.34	[0.32, 0.36]	0.30	[0.28, 0.32]	0.34	[0.31, 0.36]
Same or both-gender attracted	0.41	[0.38, 0.43]	0.33	[0.30,0.36]	0.35	[0.30, 0.40]
*Social victimization*						
Other-gender attracted	0.30	[0.29, 0.32]	0.27	[0.25, 0.28]	0.30	[0.28, 0.32]
Same or both-gender attracted	0.36	[0.33, 0.39]	0.32	[0.30, 0.34]	0.34	[0.30, 0.37]
*Light physical victimization*						
Other-gender attracted	0.27	[0.25, 0.28]	0.25	[0.23, 0.26]	0.31	[0.28, 0.33]
Same or both-gender attracted	0.32	[0.29, 0.35]	0.27	[0.25, 0.30]	0.33	[0.29, 0.38]
*Heavy physical victimization*						
Other-gender attracted	0.19	[0.17, 0.21]	0.17	[0.16, 0.19]	0.16	[0.14, 0.19]
Same or both-gender attracted	0.24	[0.22, 0.27]	0.20	[0.18, 0.23]	0.16	[0.13, 0.20]
*Sexual physical victimization*						
Other-gender attracted	0.10	[0.09, 0.11]	0.09	[0.08, 0.10]	0.12	[0.10, 0.13]
Same or both-gender attracted	0.18	[0.16, 0.20]	0.15	[0.13, 0.16]	0.16	[0.13, 0.18]
	*M*	95% CI	*M*	95% CI	*M*	95% CI
*School social unsafety*						
Other-gender attracted	1.90	[1.85, 1.95]	1.83	[1.79, 1.87]	1.76	[1.70, 1.81]
Same or both-gender attracted	2.08	[1.98, 2.17]	1.96	[1.89, 2.02]	1.86	[1.78, 1.94]
**Females**						
*Verbal victimization*						
Other-gender attracted	0.50	[0.47, 0.53]	0.44	[0.42, 0.47]	0.40	[0.36, 0.43]
Same or both-gender attracted	0.56	[0.53, 0.59]	0.52	[0.49, 0.55]	0.45	[0.40, 0.49]
*Material victimization*						
Other-gender attracted	0.25	[0.23, 0.27]	0.20	[0.19, 0.22]	0.22	[0.19, 0.25]
Same or both-gender attracted	0.30	[0.27, 0.32]	0.29	[0.26, 0.32]	0.24	[0.20, 0.28]
*Social victimization*						
Other-gender attracted	0.39	[0.37, 0.42]	0.34	[0.32, 0.36]	0.33	[0.30, 0.36]
Same or both-gender attracted	0.44	[0.41, 0.47]	0.42	[0.39, 0.44]	0.40	[0.36, 0.43]
*Light physical victimization*						
Other-gender attracted	0.20	[0.18, 0.22]	0.16	[0.15, 0.18]	0.18	[0.16, 0.21]
Same or both-gender attracted	0.26	[0.23, 0.29]	0.23	[0.21, 0.26]	0.22	[0.18, 0.26]
*Heavy physical victimization*						
Other-gender attracted	0.08	[0.07, 0.10]	0.07	[0.06, 0.09]	0.05	[0.03, 0.06]
Same or both-gender attracted	0.15	[0.13, 0.17]	0.12	[0.10, 0.14]	0.06	[0.04, 0.08]
*Sexual physical victimization*						
Other-gender attracted	0.13	[0.12, 0.15]	0.12	[0.11, 0.13]	0.14	[0.13, 0.16]
Same or both-gender attracted	0.19	[0.17, 0.22]	0.19	[0.17, 0.22]	0.19	[0.16, 0.22]
	*M*	95% CI	*M*	95% CI	*M*	95% CI
*School social unsafety*						
Other-gender attracted	1.96	[1.90, 2.02]	1.92	[1.87, 1.96]	1.83	[1.77, 1.89]
Same or both-gender attracted	2.14	[2.07, 2.20]	2.07	[2.00, 2.15]	1.94	[1.84, 2.03]

**Table 3:** Trends in Bullying Victimization and School Social Unsafety across Years by Gender Modality

	2014		2016		2018	
	Proportion	95% CI	Proportion	95% CI	Proportion	95% CI
*Verbal victimization*						
Cisgender	0.53	[0.51, 0.55]	0.49	[0.46, 0.51]	0.47	[0.44, 0.50]
Gender diverse	0.65	[0.61, 0.69]	0.64	[0.61, 0.67]	0.59	[0.53, 0.66]
*Material victimization*						
Cisgender	0.30	[0.28, 0.31]	0.26	[0.24, 0.28]	0.28	[0.26, 0.30]
Gender diverse	0.43	[0.39, 0.46]	0.40	[0.37, 0.43]	0.38	[0.31, 0.46]
*Social victimization*						
Cisgender	0.35	[0.33, 0.36]	0.30	[0.29, 0.32]	0.32	[0.30, 0.34]
Gender diverse	0.51	[0.47, 0.56]	0.48	[0.44, 0.51]	0.48	[0.41, 0.54]
*Light physical victimization*						
Cisgender	0.23	[0.22, 0.25]	0.21	[0.20, 0.23]	0.25	[0.23, 0.27]
Gender diverse	0.39	[0.35, 0.43]	0.35	[0.32, 0.38]	0.35	[0.29, 0.41]
*Heavy physical victimization*						
Cisgender	0.14	[0.12, 0.15]	0.13	[0.11, 0.14]	0.10	[0.09, 0.12]
Gender diverse	0.30	[0.27, 0.32]	0.24	[0.21, 0.27]	0.19	[0.13, 0.25]
*Sexual physical victimization*						
Cisgender	0.11	[0.10, 0.12]	0.11	[0.10, 0.12]	0.13	[0.12, 0.15]
Gender diverse	0.34	[0.31, 0.38]	0.28	[0.25, 0.31]	0.29	[0.22, 0.35]
	*M*	95% CI	*M*	95% CI	*M*	95% CI
*School Social unsafety*						
Cisgender	1.93	[1.88, 1.98]	1.87	[1.83, 1.91]	1.79	[1.75, 1.85]
Gender diverse	2.42	[2.31, 2.52]	2.30	[2.22, 2.38]	2.26	[2.12, 2.39]

For gender modality, mixed patterns were observed as well. For verbal, material (only for gender diverse participants), social (only for gender diverse participants), and heavy physical victimization a decline was observed over the years, as was for school social unsafety. A decline followed by an increase was observed for material (only for cisgender participants), social (only for cisgender participants), light physical, and sexual victimization (only for cisgender participants). Last, for cisgender participants an increase over the years in sexual victimization was observed.

### Differences in Bullying Victimization and School Social Unsafety

Table 4 displays sexual orientation-based differences in bullying victimization and school social unsafety stratified by sex assigned at birth by each year. Table 5 displays differences based on gender modality. For 2014, same or both-gender attracted male adolescents had higher odds of experiencing material (*OR* = 1.21; 95% CI [1.07, 1.37]), social (*OR* = 1.18; 95% CI [1.02, 1.35]), light physical (*OR* = 1.17; 95% CI [1.02, 1.34]), heavy physical (*OR* = 1.17; 95% CI [1.04, 1.32]), and sexual victimization (*OR* = 1.52; 95% CI [1.29, 1.80]) and reported higher school social unsafety (*b* = 0.11; 95% CI [0.05, 0.18]) than other-gender attracted males. Same or both-gender attracted female adolescents had higher odds of heavy physical (*OR* = 1.43; 95% CI [1.16, 1.75]) and sexual victimization (*OR* = 1.23; 95% CI [1.04, 1.44]), and reported higher school social unsafety (*b* = 0.09; 95% CI [0.05, 0.14]) than other-gender attracted females. Patterns in 2016 were different. That is, among males, only sexual attraction-based differences in social (*OR* = 1.17; 95% CI [1.04, 1.31]) and sexual victimization (*OR* = 1.52; 95% CI [1.31, 1.74]) were found as well as for school social unsafety (*b* = 0.08; 95% CI [0.04, 0.13]). For females, same or both-gender attracted adolescents reported higher odds of all forms of victimization and higher rates of school social unsafety. Last, focusing on 2018, no statistically significant sexual orientation-based differences in the odds of experiencing any type of victimization or school social unsafety among males was found. For females, only significant sexual orientation-based differences in social (*OR* = 1.28; 95% CI [1.10, 1.48]) and sexual (*OR* = 1.27; 95% CI [1.07, 1.52]) victimization were observed, as well as higher rates of school social unsafety (*b* = 0.07; 95% CI [0.003, 0.14]).

**Table 4:** Sexual Orientation-Based Disparities in Bully Victimization and School Social Unsafety within each Year

	2014		2016		2018	
	*OR*	95% CI	*OR*	95% CI	*OR*	95% CI
**Males**						
*Verbal victimization*						
Same or both-gender attracted^1^	1.15	[0.99, 1.34]	1.00	[0.91, 1.10]	1.03	[0.86, 1.24]
*Material victimization*						
Same or both- gender attracted^1^	**1.21**	[1.07, 1.37]	1.05	[0.93, 1.18]	1.03	[0.86, 1.24]
*Social victimization*						
Same or both- gender attracted^1^	**1.18**	[1.02, 1.35]	**1.17**	[1.04, 1.31]	1.09	[0.92, 1.30]
*Light physical victimization*						
Same or both- gender attracted^1^	**1.17**	[1.02, 1.34]	1.04	[0.93, 1.16]	1.04	[0.84, 1.29]
*Heavy physical victimization*						
Same or both- gender attracted^1^	**1.17**	[1.04, 1.32]	1.07	[0.94, 1.20]	0.90	[0.69, 1.19]
*Sexual victimization*						
Same or both- gender attracted^1^	**1.52**	[1.29, 1.80]	**1.52**	[1.31, 1.74]	1.27	[1.00, 1.61]
	*b*	95% CI	*b*	95% CI	*b*	95% CI
*School social unsafety*						
Same or both- gender attracted^1^	**0.11**	[0.05, 0.18]	**0.08**	[0.04, 0.13]	0.06	[−0.01, 0.14]
**Females**						
*Verbal victimization*						
Same or both- gender attracted^1^	1.11	[0.99, 1.24]	**1.18**	[1.06, 1.31]	1.14	[0.96, 1.35]
*Material victimization*						
Same or both- gender attracted^1^	1.10	[0.97, 1.24]	**1.35**	[1.18, 1.55]	1.02	[0.86, 1.23]
*Social victimization*						
Same or both- gender attracted^1^	1.06	[0.93, 1.20]	**1.20**	[1.06, 1.34]	**1.28**	[1.10, 1.48]
*Light physical victimization*						
Same or both- gender attracted^1^	1.15	[0.98, 1.34]	**1.30**	[1.13, 1.49]	1.16	[0.96, 1.40]
*Heavy physical victimization*						
Same or both- gender attracted^1^	**1.43**	[1.16, 1.75]	**1.32**	[1.13, 1.54]	0.95	[0.68, 1.34]
*Sexual victimization*						
Same or both- gender attracted^1^	**1.23**	[1.04, 1.44]	**1.48**	[1.28, 1.70]	**1.27**	[1.07, 1.52]
	*b*	95% CI	*b*	95% CI	*b*	95% CI
*School social unsafety*						
Same or both- gender attracted^1^	**0.09**	[0.05, 0.14]	**0.09**	[0.04, 0.13]	**0.07**	[0.003, 0.14]

*Notes*. Adjusted for level of education, grade, migration background, and gender modality

Bold numbers indicate significance at *p* < 0.05

^1^Reference group = Other-gender attracted

**Table 5:** Gender Modality-Based Disparities in Bullying Victimization and School Social Unsafety within each Year

	2014		2016		2018	
	OR	95% CI	OR	95% CI	OR	95% CI
*Verbal victimization*						
Gender diverse^1^	**1.62**	[1.35, 1.94]	**1.95**	[1.70, 2.23]	**1.69**	[1.27, 2.25]
*Material victimization*						
Gender diverse^1^	**1.72**	[1.46, 2.03]	**1.95**	[1.71, 2.22]	**1.77**	[1.28, 2.44]
*Social victimization*						
Gender diverse^1^	**1.81**	[1.55, 2.12]	**1.80**	[1.52, 2.12]	**1.67**	[1.27, 2.21]
*Light physical victimization*						
Gender diverse^1^	**2.09**	[1.78, 2.46]	**2.10**	[1.81, 2.45]	**1.67**	[1.28, 2.34]
*Heavy physical victimization*						
Gender diverse^1^	**2.51**	[2.00, 3.16]	**2.19**	[1.83, 2.61]	**2.35**	[1.41, 3.94]
*Sexual victimization*						
Gender diverse^1^	**3.34**	[2.82, 3.96]	**2.24**	[1.93, 2.60]	**1.91**	[1.40, 2.61]
	*b*	95% CI	*b*	95% CI	*b*	95% CI
*School social unsafety*						
Gender diverse^1^	**0.38**	[0.30, 0.45]	**0.34**	[0.27, 0.41]	**0.36**	[0.24, 0.47]

*Notes*. Adjusted for level of education, grade, migration background, sexual orientation, and sex assigned at birth

Bold numbers indicate significance at *p* < 0.05

^1^Reference group = cisgender

Concerning gender modality-based disparities, across all years, gender diverse adolescents had higher odds of reporting all forms of bullying victimization and school social unsafety when compared to their cisgender peers.

### Trends in Bullying Victimization and School Social Unsafety

Table 6 presents sexual orientation and gender modality-based trends in bullying victimization and school social unsafety. Focusing on sexual orientation first, no changes were observed among males. That is, sexual orientation-based disparities in verbal, material, social, light physical, heavy physical, and sexual victimization did not change in their relative size between 2014 and 2016 and between 2014 and 2018. Neither did sexual orientation-based disparities in school social unsafety. For females, sexual orientation-based disparities in verbal, social, light physical, heavy physical, and sexual victimization did not change in their relative size between 2014 and 2016 and between 2014 and 2018. Neither did sexual orientation-based disparities in school social unsafety. The exception was that sexual orientation-based disparities in material victimization among females widened between 2014 and 2016 (*OOR* = 1.29; 95% CI [1.09, 1.51]).

**Table 6:** Sexual Orientation and Gender Modality-Based Trends in Bully Victimization and School Social Unsafety

	Verbal victimization		Material victimization		Social victimization		Light physical victimization		Heavy physical victimization		Sexual victimization		School social unsafety	
	*OOR*^1^	95% CI	*OOR*^1^	95% CI	*OOR*^1^	95% CI	*OOR*^1^	95% CI	*OOR*^1^	95% CI	*OOR*^1^	95% CI	*b*	95% CI
**Males**														
Same or both-gender attracted × 2016^2^	0.90	[0.76, 1.07]	0.85	[0.73, 1.00]	1.01	[0.85, 1.21]	0.90	[0.76, 1.06]	0.91	[0.76, 1.07]	0.95	[0.78, 1.17]	−0.03	[−0.11, 0.05]
Same or both-gender attracted × 2018^2^	0.94	[0.76, 1.19]	0.84	[0.69, 1.03]	0.97	[0.78, 1.20]	0.91	[0.71, 1.16]	0.79	[0.58, 1.08]	0.80	[0.60, 1.06]	−0.04	[−0.13, 0.06]
**Females**														
Same or both-gender attracted × 2016^2^	1.08	[0.94, 1.26]	**1.29**	[1.09, 1.51]	1.13	[0.97, 1.31]	1.12	[0.94, 1.34]	0.92	[0.72, 1.18]	1.11	[0.92, 1.34]	−0.02	[−0.08, 0.05]
Same or both-gender attracted × 2018^2^	1.02	[0.84, 1.26]	0.94	[0.75, 1.18]	1.17	[0.96, 1.43]	0.97	[0.76, 1.23]	0.66	[0.45, 0.98]	0.93	[0.74, 1.18]	−0.03	[−0.11, 0.05]
**Gender modality**														
Gender diverse × 2016^3^	1.16	[0.94, 1.43]	1.12	[0.93, 1.34]	1.00	[0.82, 1.23]	0.98	[0.79, 1.22]	0.88	[0.70, 1.12]	**0.76**	[0.61, 0.93]	−0.06	[−0.16, 0.03]
Gender diverse × 2018^3^	0.97	[0.72, 1.31]	0.89	[0.64, 1.25]	0.91	[0.66, 1.21]	0.75	[0.54, 1.03]	0.74	[0.48, 1.16]	**0.60**	[0.43, 0.85]	−0.04	[−0.19, 0.11]

*Notes*. Adjusted for level of education, grade, migration background, gender modality (only in models with sexual orientation as predictor), sexual orientation (only in models with gender modality as predictor), and sex assigned at birth (only in models with gender modality as predictor)

Bold numbers indicate significance at *p* < 0.05

^1^OOR = Odds of odds ratio

^2^Reference group = Other-gender attracted * 2014

^3^Reference group = Cisgender * 2014

Gender modality-based disparities in sexual victimization narrowed between 2014 and 2016 (*OOR* = 0.76; 95% CI [0.61, 0.93]) and between 2014 and 2018 (*OOR* = 0.60; 95% CI [0.43, 0.85]). Disparities in verbal, material, social, light physical, and heavy physical victimization did not change in their relative size between 2014 and 2016 and between 2014 and 2018. Neither did sexual modality-based disparities in school social unsafety.

## Discussion

Research on trends in bullying victimization disparities is US focused, does not consider trends of social unsafety in schools, and has only focused on sexual orientation-based disparities and not on gender modality-based disparities. To overcome these limitations of past research, the current study examined trends in both bullying victimization and social unsafety in schools for sexually and gender diverse adolescents in the Netherlands.

It was expected that sexually diverse adolescents would report more bullying victimization and feelings of school social unsafety than heterosexual adolescents. However, the size of sexual orientation-based disparities in bullying victimization and feelings of unsafety in school decreased with each year, especially among adolescent males (for an overview, see Table 7). This is contrary to research that identified sexual orientation-based disparities in bullying victimization (Martin-Storey & Fish, 2019) and school social unsafety (Kosciw et al., 2022). Further, it was also expected that sexual orientation-based disparities in bullying victimization and school social unsafety would remain stable over time. Results indicated that sexual orientation-based disparities in bullying victimization and school social unsafety indeed remained stable. This was also in line with previous research on trends in bullying victimization (Goodenow et al., 2016; Poteat et al., 2019). There was one exception, among females, sexual orientation-based disparities in material victimization (e.g., belongings that were damaged) widened between 2014 and 2016.

**Table 7:** Overview of the Findings

	2014	2016	2018	Trends 2014–2016	Trends 2014–2018
**Sexual orientation-based disparities among males**					
Verbal victimization	Ns	Ns	Ns	Ns	Ns
Material victimization	+	Ns	Ns	Ns	Ns
Social victimization	Ns	+	Ns	Ns	Ns
Light physical victimization	+	Ns	Ns	Ns	Ns
Heavy physical victimization	+	Ns	Ns	Ns	Ns
Sexual victimization	+	+	Ns	Ns	Ns
School social unsafety	+	+	Ns	Ns	Ns
**Sexual orientation-based disparities among females**					
Verbal victimization	Ns	+	Ns	Ns	Ns
Material victimization	Ns	+	Ns	+	Ns
Social victimization	Ns	+	+	Ns	Ns
Light physical victimization	Ns	+	Ns	Ns	Ns
Heavy physical victimization	+	+	Ns	Ns	Ns
Sexual victimization	+	+	+	Ns	Ns
School social unsafety	+	+	Ns	Ns	Ns
**Gender modality-based disparities**					
Verbal victimization	+	+	+	Ns	Ns
Material victimization	+	+	+	Ns	Ns
Social victimization	+	+	+	Ns	Ns
Light physical victimization	+	+	+	Ns	Ns
Heavy physical victimization	+	+	+	Ns	Ns
Sexual victimization	+	+	+	-	-
School social unsafety	+	+	+	Ns	Ns

Notes. “+” indicates for each year whether sexually or gender diverse adolescents had higher odds of bullying victimization or higher rates of school social unsafety than their heterosexual or cisgender peers. For trends over years, it indicates whether disparities have widened

“-” indicates for each year whether sexually or gender diverse adolescents had lower odds of bullying victimization or lower rates of school social unsafety than their heterosexual or cisgender peers. For trends over years, it indicates whether disparities have narrowed

“Ns” indicates that no significant differences were found

The findings concerning the stability in sexual orientation-based trends in bullying victimization and school social unsafety over time are consistent with predictions of the developmental collision hypothesis, although the underlying mechanisms were not examined. That is, the present study’s stable trends of sexual orientation-based differences in bullying victimization and school social unsafety could be explained by the improvement of acceptance of sexual diversity (Huijnk, 2022), resulting in adolescents coming out during a developmental period during which peer social regulation and the policing of gender expression and heteronormative behavior peaks (Russell & Fish, 2019). Thus, because contemporary sexually and gender diverse adolescents come out at a young age, they remain vulnerable to bullying victimization and feel unsafe in schools, resulting in stable trends.

Within each year, inconsistent sexual orientation-based disparities in bullying victimization and feelings of school social unsafety were found. That is surprising considering that research previously identified sexual orientation-based differences in bullying victimization (la Roi et al., 2016) and feelings of unsafety (Mooij, 2016; Scholte et al., 2016) in the Netherlands. It is also surprising because sexually diverse Dutch adolescents report poorer mental health than their heterosexual peers (Kiekens et al., 2023), which is hypothesized to stem from experiences with, for instance, victimization (Meyer, 2003). One potential explanation for the inconsistent results concerning bullying victimization is the use of single-item measures in previous studies (la Roi et al., 2016) and the measurement of several forms of bullying victimization in the present study. Possibly, when single-item measures are used more prominent disparities are observed because such measures capture a broader set of bullying behaviors compared to separately measuring multiple forms of bullying victimization. This explanation does not hold for school social unsafety, especially because a study using the same measure found clearer disparities (Scholte et al., 2016). Further, the finding that same or both-gender attracted adolescents might report similar levels of bullying victimization as their other gender-attracted peers does not mean that they are not affected by stigma. They might still anxiously expect rejection to happen, inside or outside their school environment (Kiekens et al., 2022), or face less overt forms of stigma such as microaggressions (Kiekens et al., 2022), which both negatively affect mental health (Kaufman et al., 2017).

Focusing on gender modality, it was expected that gender diverse adolescents would report more bullying victimization and feelings of school social unsafety than cisgender adolescents. Findings indicated that across all survey cycles, gender diverse adolescents reported higher rates of bullying victimization and school social unsafety compared to cisgender adolescents. It was also expected that gender modality-based disparities in bullying victimization and school social unsafety remained stable over time. The results showed that gender modality-based disparities in bullying victimization and school social unsafety remained stable, except for sexual victimization where disparities became smaller (or narrowed), between 2014 and 2016 and 2014 and 2018. All in all, these findings suggest that gender diverse adolescents were more likely to experience bullying victimization and feelings of school social unsafety, and that these disparities remained stable over time, which was consistent with the present study’s expectations.

The findings regarding gender modality add to the literature in two important ways. First, previous research found stable sexual orientation-based trends in bullying victimization (Goodenow et al., 2016; Poteat et al., 2019) but no research has examined this for gender modality-based disparities in bullying victimization. This study is the first to extend these findings to gender diverse adolescents, also concerning feelings of school social unsafety. This echoes cross-sectional research that shows that gender diverse adolescents are more likely to encounter stigma in schools and further identifies that these disparities have not waned in the context of (presumed) social progress for transgender and gender diverse adolescents (Delozier et al., 2020; Martín-Castillo et al., 2020). Second, the developmental collision hypothesis (Russell & Fish, 2019) was developed to explain the confluences of contextual and developmental processes through which contemporary adolescents continue to experience elevated risk for poor mental and behavioral health. The present study is consistent with tenants of this hypothesis and further suggests that these same processes might also contribute to poor outcomes for gender diverse adolescents.

The present study indicates that sexual orientation- and gender modality-based trends in bullying victimization and feelings of school social unsafety have remained generally stable in the Netherlands between 2014 and 2018. That is: for same- or both-gender attracted adolescents, disparities have remained relatively small over time, whereas for gender diverse adolescents, large disparities were found for each survey cycle. This indicates that deviation from (binary) gender norms increases the risk of bullying victimization and feeling unsafe in schools, rather than deviating from heterosexual attractions. It is therefore important that school policies focus more on gender diversity and the stigmatization that gender diverse adolescents face. One way this can be done is with the facilitation of gender and sexuality alliances in school, which are student-initiated extracurricular school clubs for sexually and gender diverse students and allies that provide social support and opportunities for school advocacy (Baams & Russell, 2021).

This study’s findings should be interpreted considering some limitations. First, the measure of gender modality was limited as items only assessed whether a participant identified as a boy or a girl and it did not assess the level that students disclosed or expressed their gender modality. The lack of comprehensive measures of gender modality is a common problem of population-based surveys and this issue should be addressed accordingly (Baams & Kaufman, 2023).

Second, a measure of romantic attraction was used to measure sexual orientation. Previous research, especially on bullying victimization, has used measures on sexual identity (Baams & Kaufman, 2023), posing some limits to the comparability of the present study’s results. Especially considering that romantic attraction and sexual identity do not always overlap (Fish & Krueger, 2020). Thus, the implication of using a romantic attraction measure is that there is a limited comparability between findings of the present study and previous research. Generalizations should therefore be made with caution. Further, it was also not assessed to what extent students disclosed their sexual orientation to others.

Third, the operationalization of sexual orientation was based on adolescents’ sex assigned at birth. It is, therefore, possible that, for instance, someone who identifies as a transgender boy who is only attracted to boys was wrongly categorized as other-gender attracted. However, because gender identity was not assessed, it was hard to completely rule out these misspecifications of sexual attraction. This further emphasizes the need for population-based surveys to use comprehensive measures of sexual and gender identity (Temkin et al., 2017). Related to this, a drop was observed in the number of gender diverse adolescents in 2018. Future Dutch studies should closely monitor whether this is a persistent trend or if it might be specific to the current study (Baams & Kaufman, 2023).

Fourth, due to limited group sizes (i.e., in 2018, 46 males and 43 females reported same-gender attraction), it was not possible to study sexual orientation and gender modality-based sub-group differences in trends of bullying victimization and school social unsafety despite research pointing to potential subgroup differences (Kosciw et al., 2022).

Fifth, although for bullying victimization multiple items were used, feelings of school social unsafety was measured using a single item which might not fully capture this concept. Additionally, no measure of cyber bullying was used, despite the prominence of social media in the lives of adolescents (Pew Research Center, 2022). Further, this study only used self-reports of students. Especially concerning bullying victimization, research has stressed the importance of using multiple informants because the number of students categorized as bullying victims varies when self or peer-reports are used (Branson & Cornell, 2009).

Last, this study examined trends in bullying victimization from 2014 to 2018, a time span of four years. Examining trends in a relatively short period might explain why the present study hardly found support for trends in bullying victimization and school social unsafety. Research studying longer periods of time is therefore needed.

Future research on the developmental collision hypothesis could also focus on the role of age and developmental milestones. On the one hand, research could study whether disparities in rates of bullying victimization and school social unsafety differ by age. Prior research found that victimization decreased across adolescence and into early adulthood among sexually and gender diverse youth (Birkett et al., 2015) but has not studied this regarding school social unsafety. On the other hand, Research could directly compare rates of bullying victimizing and feelings of school social unsafety for sexually and gender diverse adolescents who came out in early adolescence with those who came out later in adolescence. Earlier sexual identity development milestones (e.g., ages of first same-sex attraction, self-identification, sexual behavior, disclosure) were associated with more sexual-orientation related victimization (Bishop, 2022), but whether this also holds for gender diverse people and feelings of social unsafety in school in unsure.

Although some research indicates that in the past decades the acceptance of sexual and gender diversity has improved in Western countries (Huijnk, 2022; ILGA Europe, 2023), recent developments might indicate a growing resistance. For instance, anti-sexual and gender diversity legislation was recently passed in Florida in the US (Izaguirre & Farrington, 2023), and anti-gender campaigns in Europe are gaining traction (Kuhar & Paternotte, 2017). These societal developments call for continuous monitoring to understand the impact on sexually and gender diverse adolescents’ health and experiences. Trend research can be especially valuable as this can substantiate how such developments impact adolescents over time.

## Conclusion

Trends in bullying victimization for sexually diverse adolescents have been studied in the US, finding stable disparities. However, whether such trends are also present in other social context and whether gender modality-based trends exist remained unknown. Further, similar trends for school social unsafety have not yet been examined. To overcome these limitations of past research, this study examined trends in both bullying victimization and social unsafety in schools for sexually and gender diverse adolescents in the Netherlands. Findings indicate that trends in bullying victimization and school social unsafety remained stable over the years. Concerning sexual orientation, this meant that disparities remained relatively small over time, while gender diverse adolescents remained to be more likely to be victimized and feel unsafe in school. These results imply that especially gender diverse adolescents remain vulnerable to be bully victims and feel unsafe in school and underline the importance of school policies that to foster a safe environment for adolescents that deviate from traditional gender norms.
